# Supplementary material for: Global forestation and deforestation affect remote climate via adjusted atmosphere and ocean circulation
Source: Nat Commun. 2022 Oct 4;13:5569. doi: 10.1038/s41467-022-33279-9 (PMC9532392; doi:10.1038/s41467-022-33279-9)
Supplement: Supplementary file 1 — Supplementary Information [file 41467_2022_33279_MOESM1_ESM.pdf]

Global forestation and deforestation affect remote climate via adjusted atmosphere and ocean circulation

Raphael Portmann[1,2]*, Urs Beyerle[1], Edouard Davin[1,3],
Erich M. Fischer[1], Steven De Hertog[4], Sebastian Schemm[1]

*raphael.portmann@alumni.ethz.ch

[1] Institute for Atmospheric and Climate Science, ETH Zurich, Zurich, Switzerland

[2] Present address: Agroscope Reckenholz, Climate and Agriculture,
Division of Agroecology and Environment, Zürich, Switzerland

[3] Present address: Wyss Academy for Nature, Climate and Environmental Physics,
Oeschger Centre for Climate Change Research, University of Bern, Bern, Switzerland

[4] Department of Hydrology and Hydraulic Engineering, Vrije Universiteit Brussel, Brussels, Belgium

Supplementary Information

Contents

1 Supplementary Figures **2**
1.1 Changes of land surface, albedo, radiative forcing, and near-surface climate 2
1.2 Heat transport and AMOC changes 9
1.3 Extratropical atmospheric circulation changes 14
1.4 Hadley cell strength, ITCZ shift, and the subtropical jet stream 18

2 Supplementary Methods **20**
2.1 Atlantic meridional overturning circulation in potential density coordinates 20
2.2 Computation of ocean heat content 20
2.3 Computation of the Rossby wave source 20

Supplementary References **20**

1 Supplementary Figures

1.1 Changes of land surface, albedo, radiative forcing, and near-surface climate

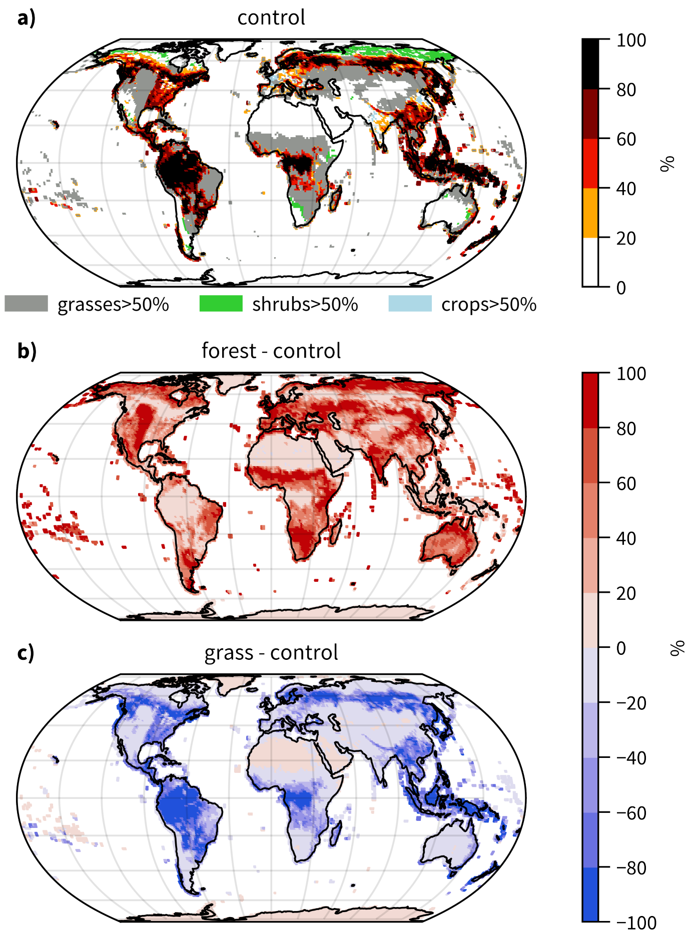

Supplementary Figure 1: Changes in land surface boundary conditions. Land area fraction (shading, in %) that is (a) covered with forest in the *control* run, (b) afforested in the *forest* run (*forest* minus *control*) and (c) deforested in the *grass* run (*grass* minus *control*). In (a), grid points where more than 50 % of the land area in *control* is covered with grass (grey), shrubs (green), and crops (blue) in *control*. This Figure is produced using Cartopy which is licensed under the GNU Lesser General Public License.

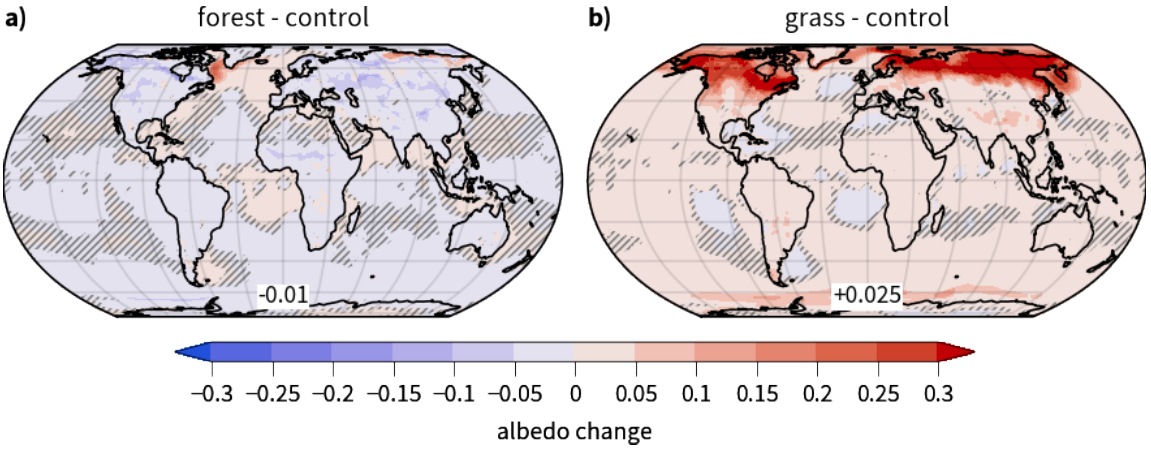

Supplementary Figure 2: Albedo changes. Differences in annual mean clear sky albedo at the Earth's surface relative to the preindustrial control run for (a) forest (b) grass. Statistically insignificant differences are hatched (see methods section). This Figure is produced using Cartopy which is licensed under the GNU Lesser General Public License.

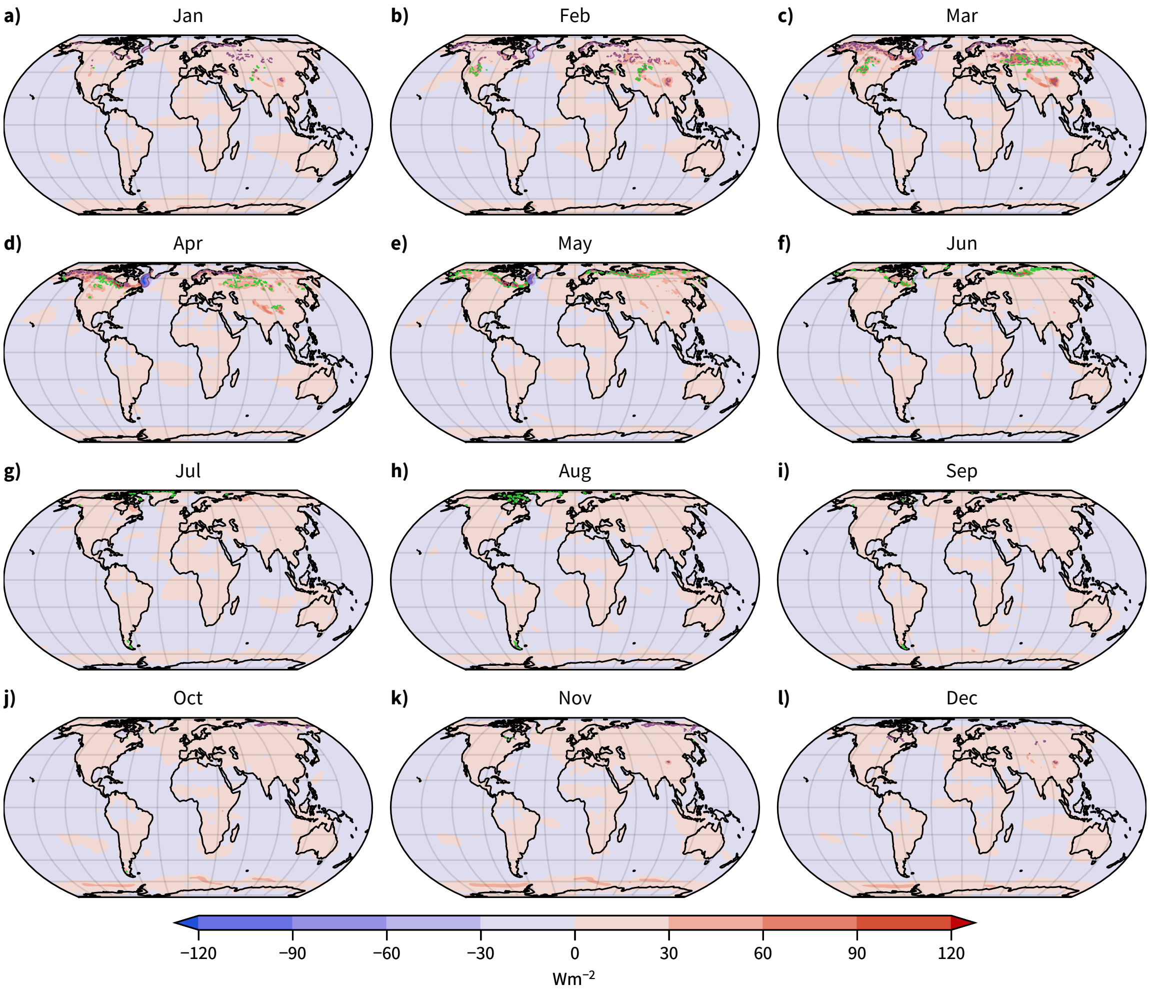

Supplementary Figure 3: No strong snow or ice albedo feedback in *forest*. Differences in monthly mean surface radiative forcing (shading), clear sky surface albedo (purple contours, -0.3 and $+0.3$), and snow cover fraction (green contours, -25% and $+25\%$) for *forest-control*. Panels (a-l) correspond to the individual months (January to December). This Figure is produced using Cartopy which is licensed under the GNU Lesser General Public License.

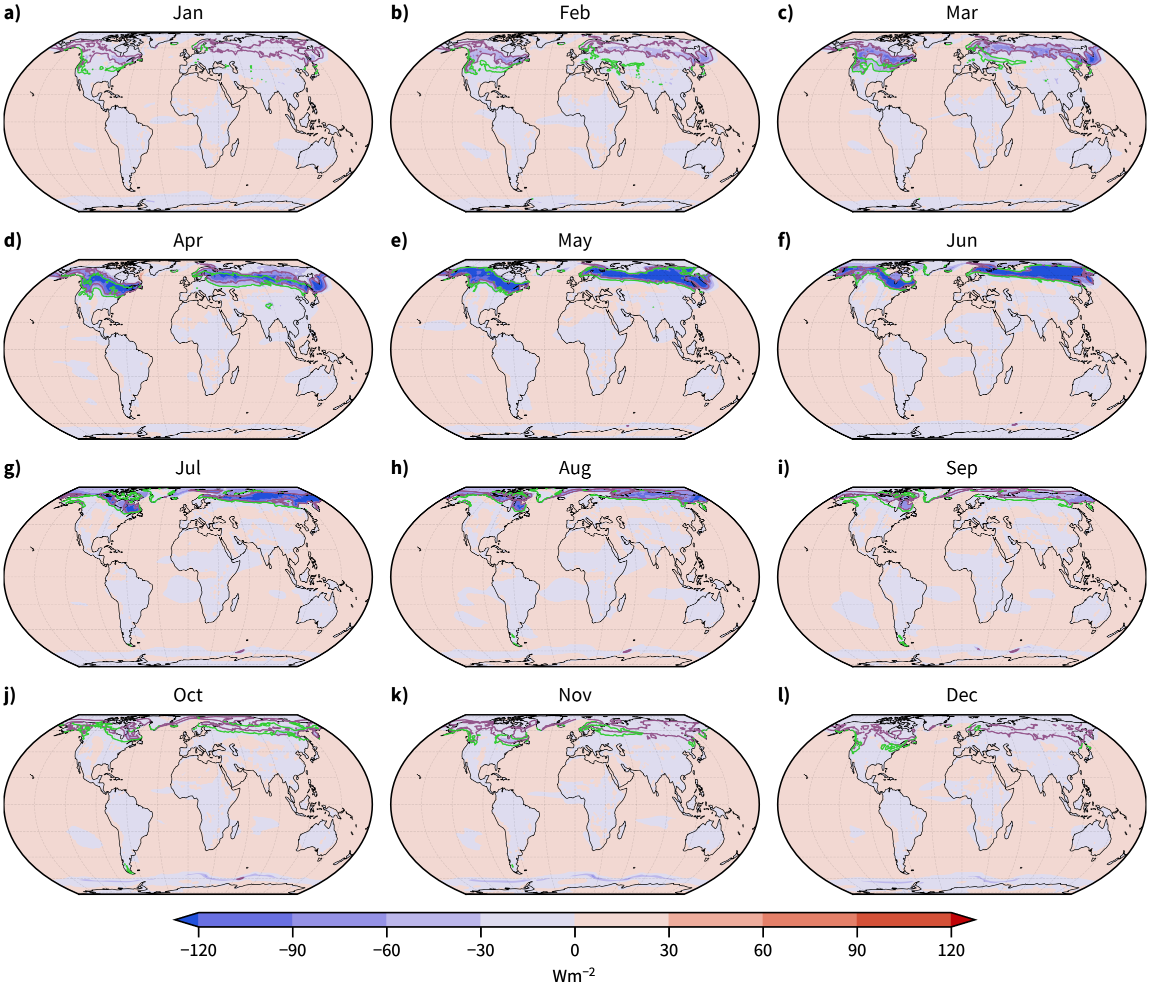

Supplementary Figure 4: Strong high-latitude snow and ice albedo feedback in *grass*. Difference in monthly mean surface radiative forcing (shading), clear sky surface albedo (purple contour, -0.3 and $+0.3$), and snow cover fraction (-25% and +25%) for *grass-control*. Panels (a-l) correspond to the individual months (January to December). This Figure is produced using Cartopy which is licensed under the GNU Lesser General Public License.

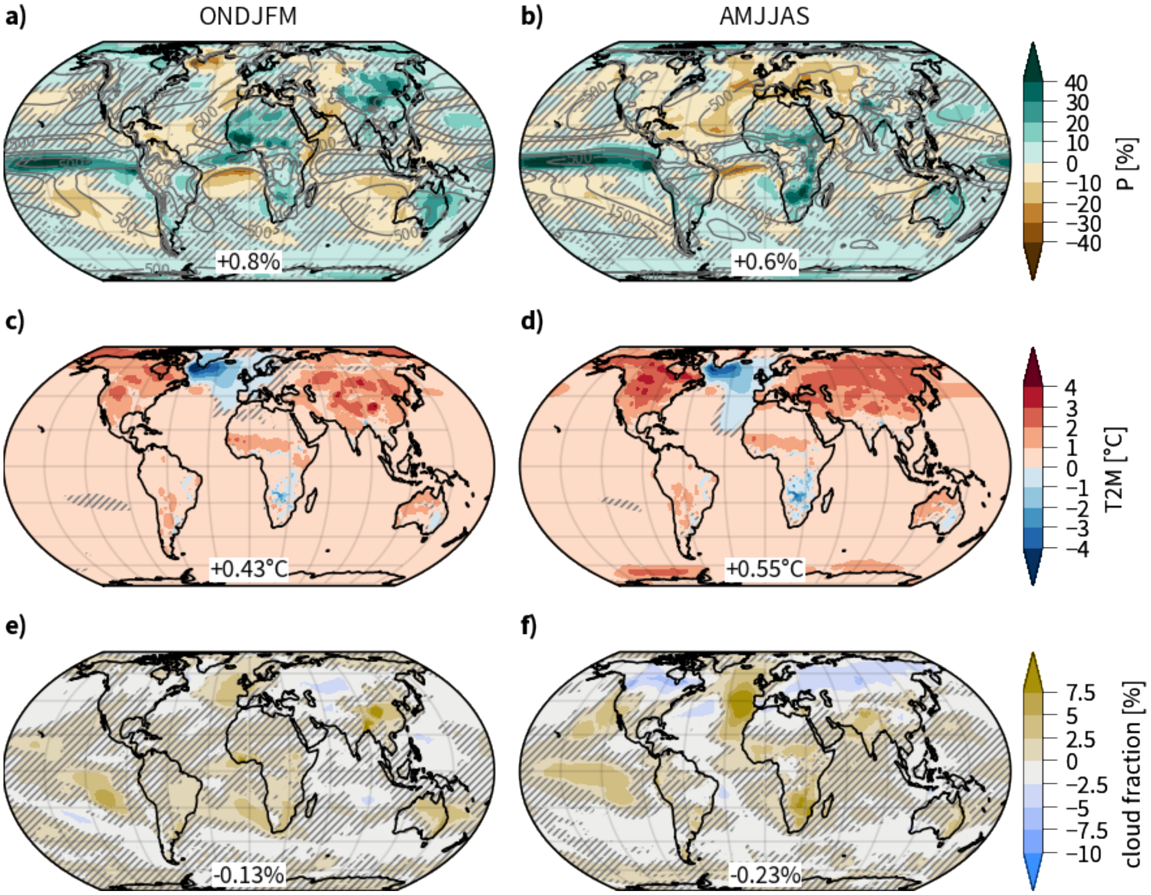

Supplementary Figure 5: Seasonal changes in key climate variables in *forest*. Seasonal mean differences between forest and control for (a,b) precipitation (relative differences), (c,d) 2 m temperature, and (e,f) total cloud fraction. Shown are means from (left column) October to March and (right column) April to September. Statistically insignificant differences are hatched (see methods section). This Figure is produced using Cartopy which is licensed under the GNU Lesser General Public License.

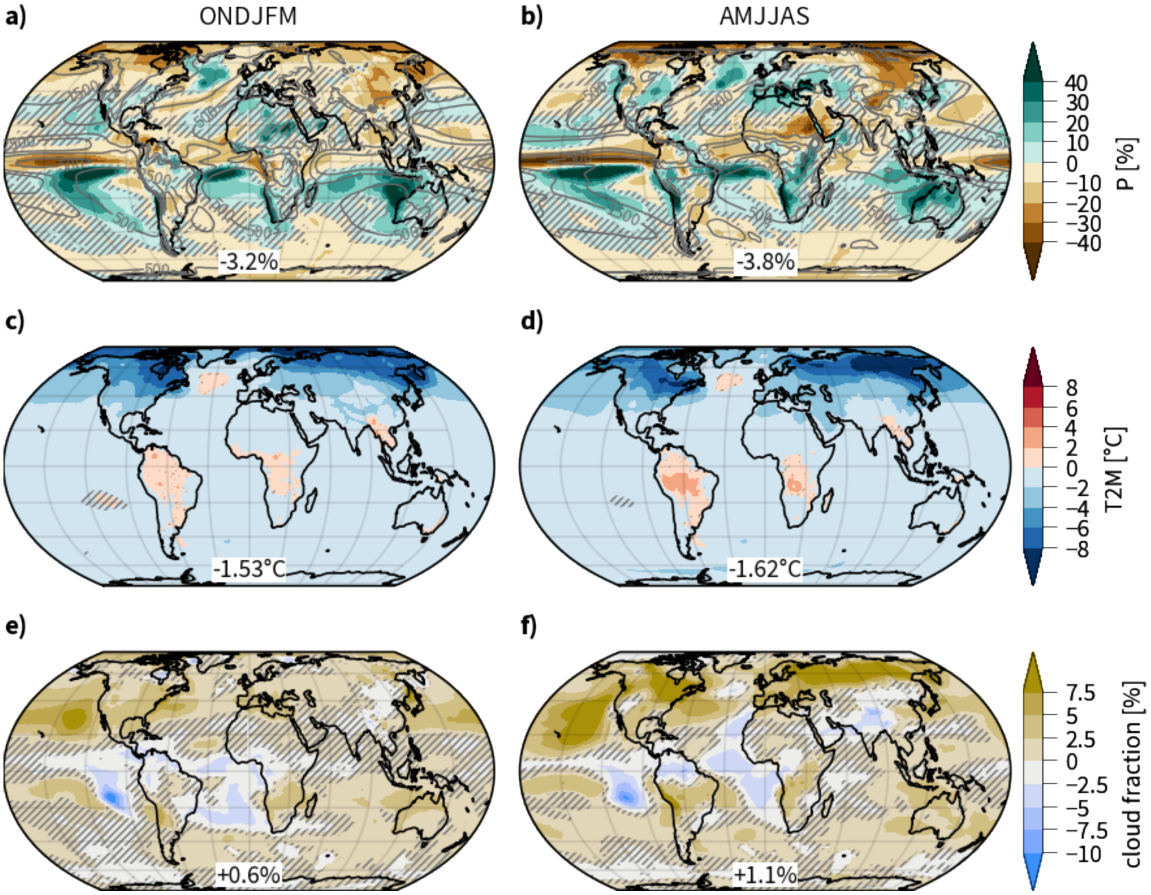

Supplementary Figure 6: Seasonal changes in key climate variables in *grass*. Seasonal mean differences between grass and control for (a,b) precipitation (relative differences), (c,d) 2 m temperature, and (e,f) total cloud fraction. Shown are means from (left column) October to March and (right column) April to September. Statistically insignificant differences are hatched (see methods section). This Figure is produced using Cartopy which is licensed under the GNU Lesser General Public License.

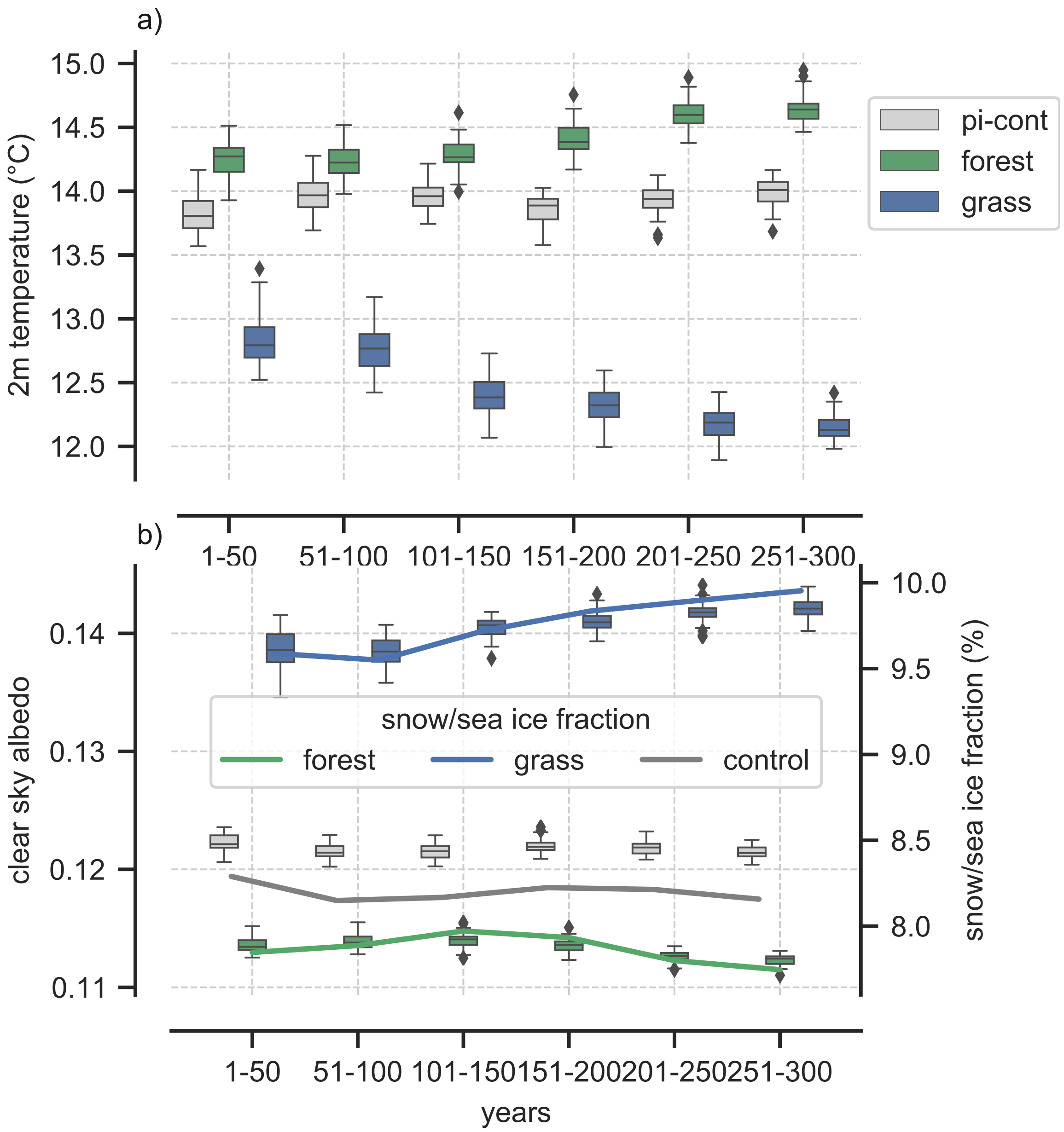

Supplementary Figure 7: Temporal evolution of global mean temperatures and albedo. Standard box plots for different time periods in the three simulations showing the temporal evolution of global mean (a) 2 m temperature and (b) clear sky albedo. In (b), additionally the 50-year mean percentage of the Earth's surface covered with sea ice or snow is shown (lines).

1.2 Heat transport and AMOC changes

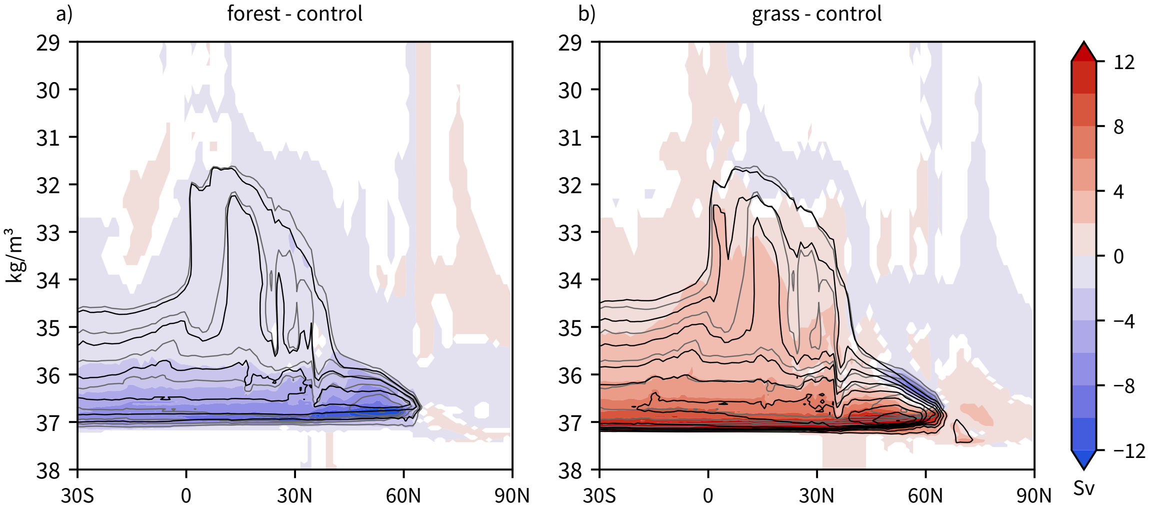

Supplementary Figure 8: Atlantic meridional overturning circulation in potential density coordinates. Shown is the annual mean meridional mass stream function in the Atlantic in potential density coordiniates, i.e. the σ-AMOC (contours, in Sv, only values above 4 Sv shown with a 4 Sv interval) for (a) forest (black) and control (gray) and (b) grass (black) and control (gray), as well as differences of experiments relative to control (shading, in Sv). Statistically insignificant differences are shown in white, see methods section.

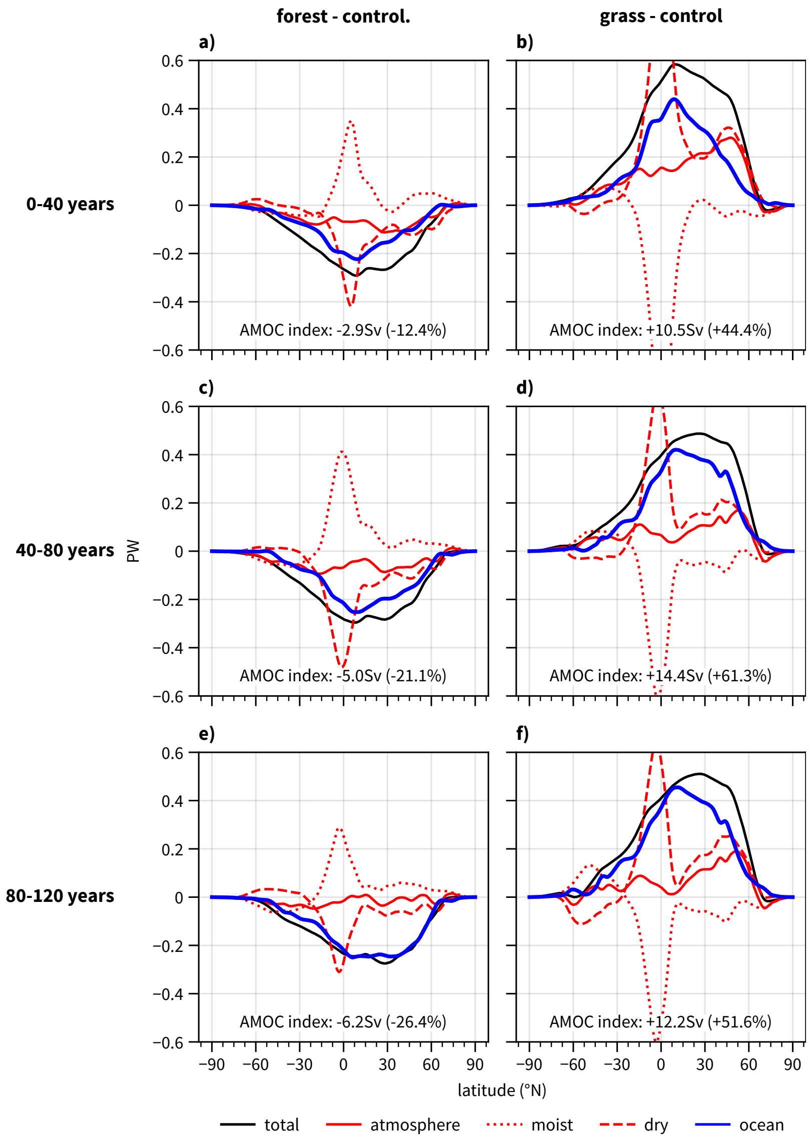

Supplementary Figure 9: Changes in meridional heat transport components in 40-year time windows. Differences of the annual mean meridional heat transport over 40-year time intervals during the first 120 years of simulation (a,c,e) *forest*, and (b,d,f) *grass* with respect to the average of annual mean transport during the first 40 years in simulation *control*. The total heat transport (black) is separated into atmospheric (red) and ocean (blue) transport. Atmospheric heat transport is further split into moist (dotted) and dry (dashed) heat transport.

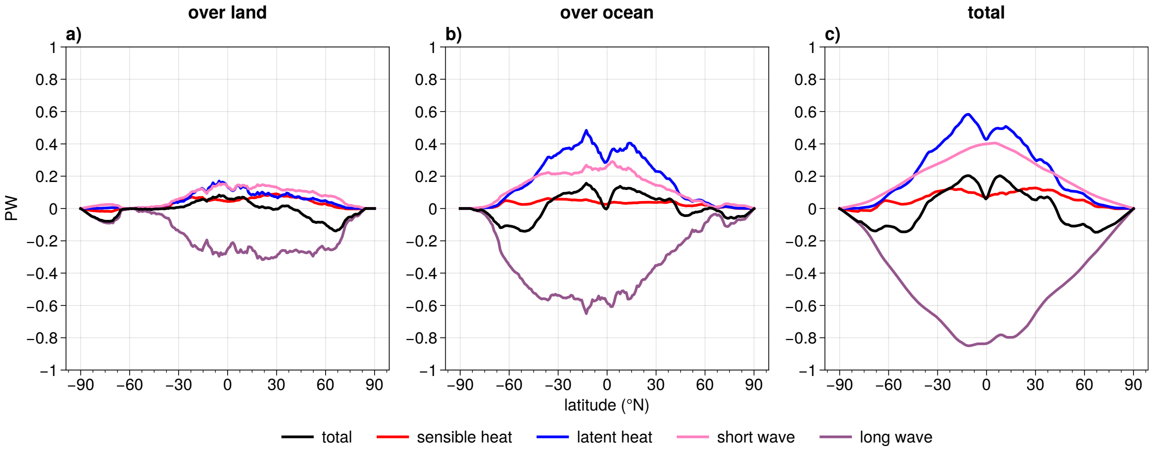

Supplementary Figure 10: Climatological atmospheric energy budget in *control*. Climatological mean components of the atmospheric energy budget for *control* at a given latitude for (a) fluxes over land, (b) fluxes over the ocean, and (c) total fluxes. Negative values indicate net energy loss from the atmospheric column and positive values a net energy gain.

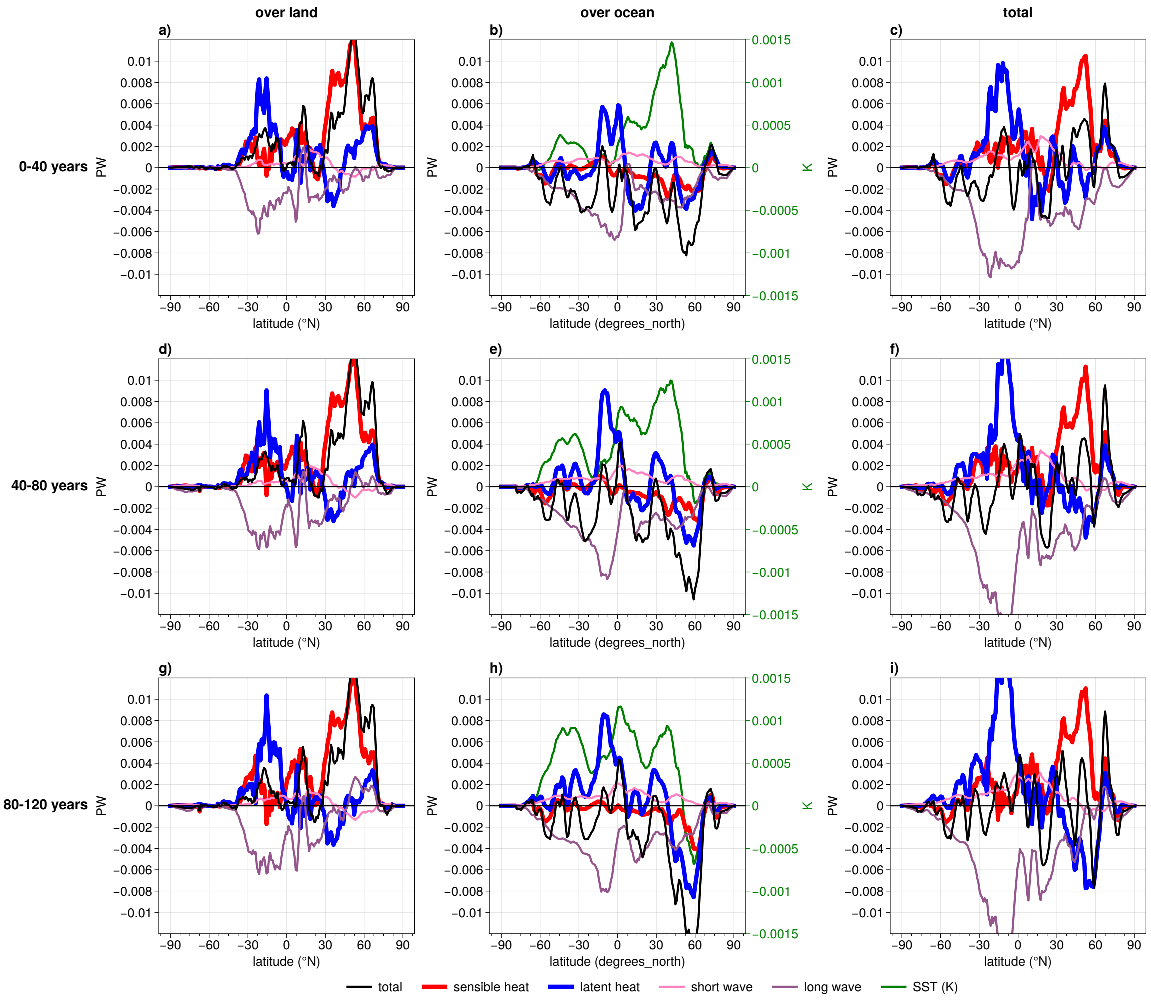

Supplementary Figure 11: Changes in the atmospheric energy budget in 40-year time windows. Absolute changes in the components of the atmospheric energy budget in *forest* at a given latitude averaged over 40-year time intervals during the first 120 years of the simulation forest with respect to the average over the first 40 years in *control* for (a,d,g) fluxes over land, (b,e,h) fluxes over the ocean, and (c,f,i) total fluxes. As a reference, the climatological mean components of the annual mean atmospheric energy budget in *control* are shown in Extended Fig. 10.

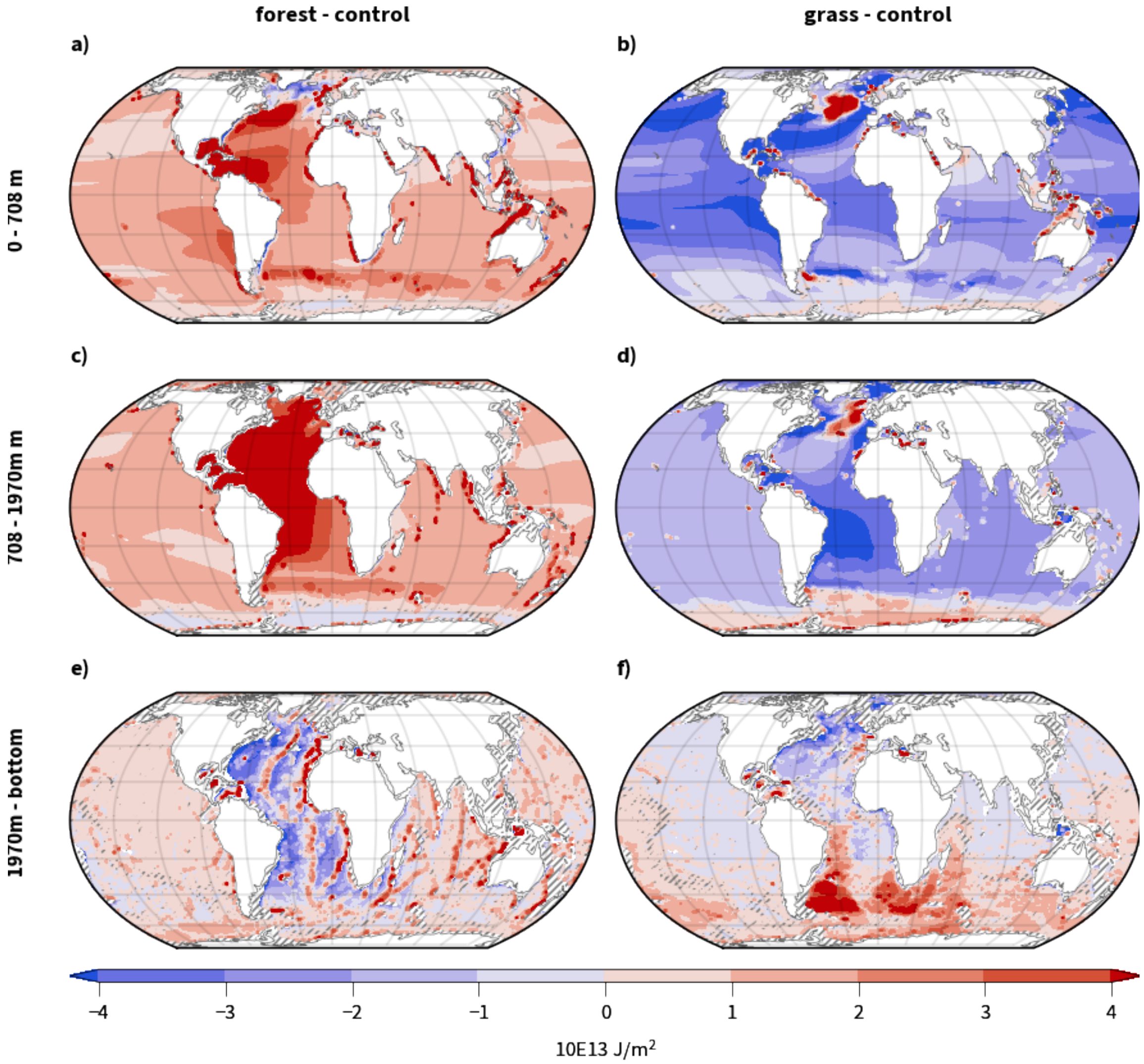

Supplementary Figure 12: Ocean heat content changes. Absolute changes of the ocean heat content (shading, in J m^{-2} from (a,b) 0-708 m, (c,d) 708-1970 m, and (e,f) 1970 m to ocean bottom in (a,c,e) *forest* and (b,d,f) *grass*. Please ignore artefacts at the ocean boundaries. This Figure is produced using Cartopy which is licensed under the GNU Lesser General Public License.

1.3 Extratropical atmospheric circulation changes

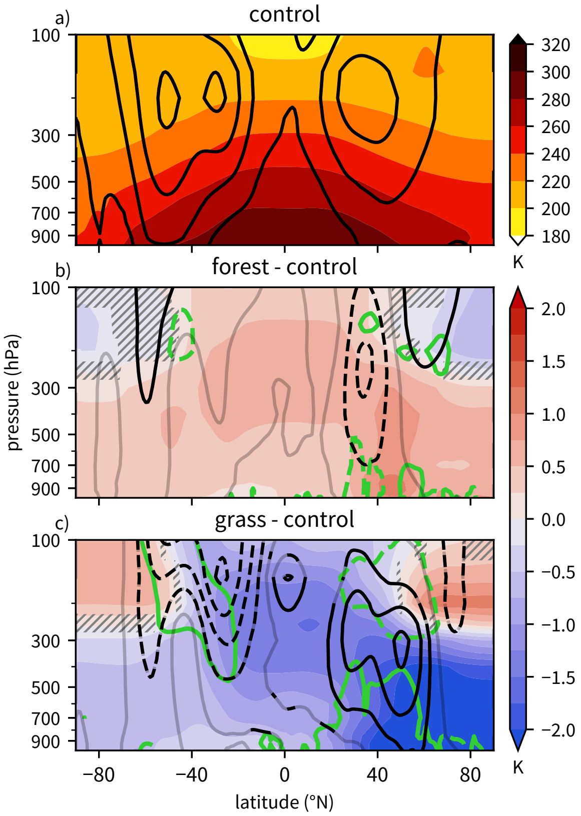

Supplementary Figure 13: Zonal mean changes in tropospheric temperature and winds aligned with thermal wind balance. Zonal mean vertical cross section of (a) annual mean temperature (shading, in K), zonal wind speed (black contours, in $\mathrm{m\,s^{-1}}$, with steps of 10 $\mathrm{m\,s^{-1}}$) for control and (b,c) differences of annual mean temperature (shading, in K, hatched where not statistically significant, see methods section) and wind speed (black contours, in $\mathrm{m\,s^{-1}}$ with steps of 0.5 $\mathrm{m\,s^{-1}}$, transparent where not statistically significant), and the meridional temperature gradient (green contours, -0.25 and 0.25 $\mathrm{K\,(10E3km)^{-1}}$) for (b) forest minus control and (c) grass minus control.

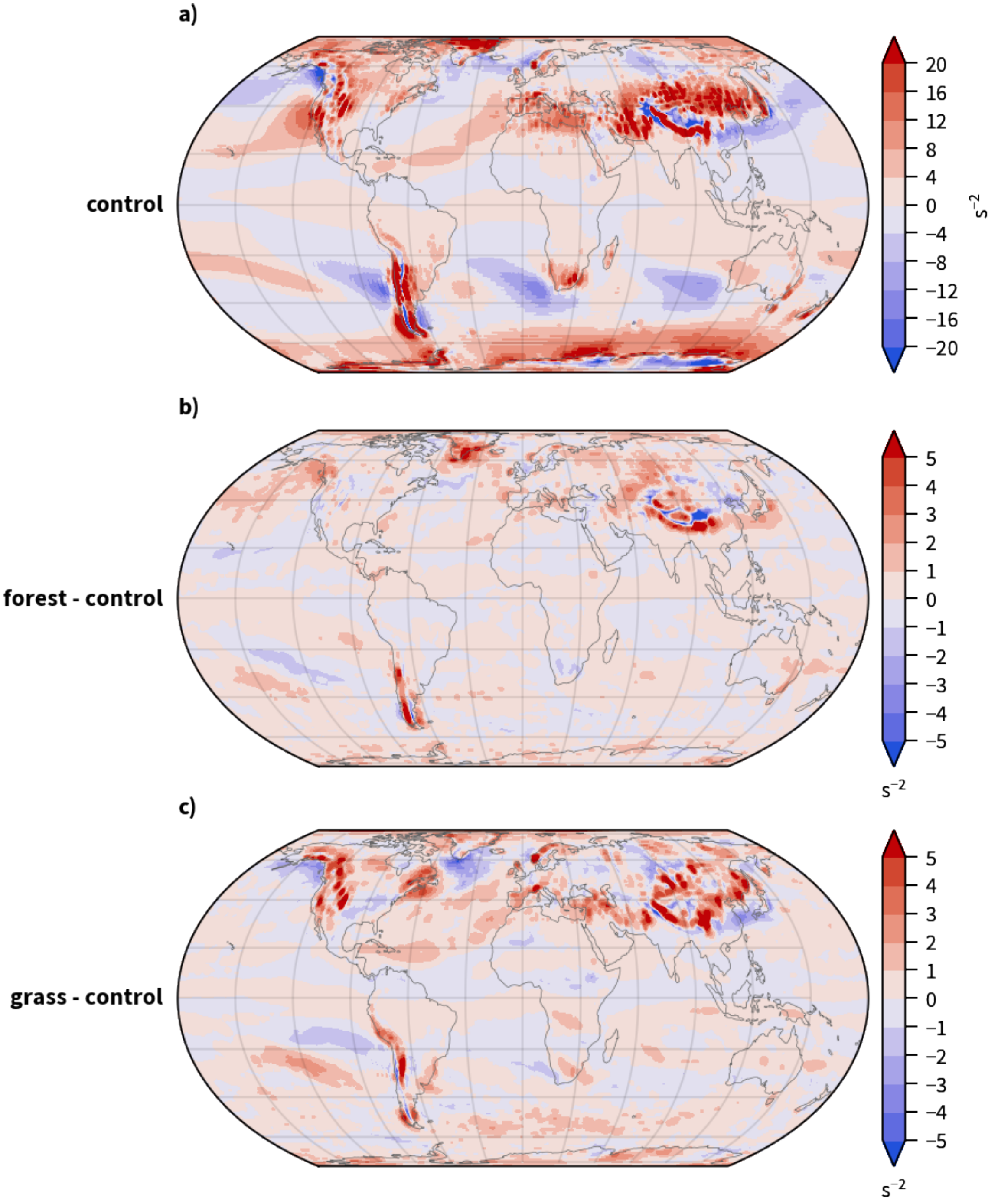

Supplementary Figure 14: No well-marked tropical sources of Rossby waves. Climatological mean Rossby wave source following [3] at 300 hPa (shading, in s^{-2}) for (a) control, (b) the difference between forest and control, and (c) the difference between grass and control. This Figure is produced using Cartopy which is licensed under the GNU Lesser General Public License.

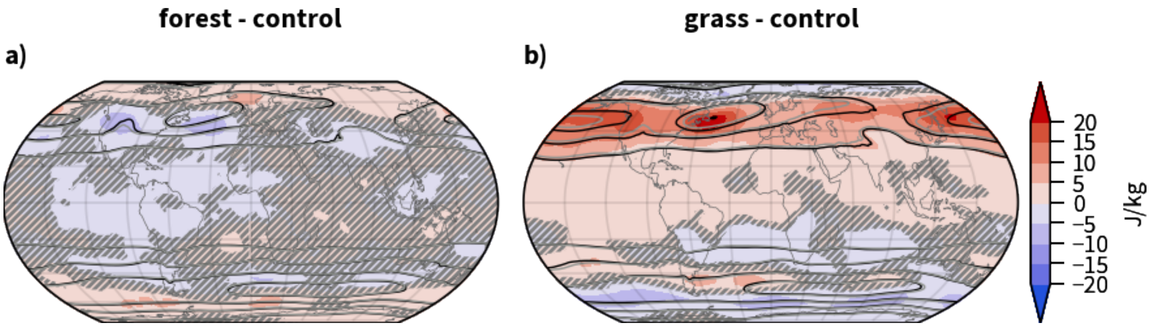

Supplementary Figure 15: Changes in intensity of extratropical eddies. Eddy kinetic energy at 300 hPa (contours, every 50 J/kg) for (a) *forest* (black) and *control* (grey), and (b) *grass* (black) and *control* (grey). Shading shows absolute differences for (a) *forest* - *control* and (b) *grass* - *control*. Statistically insignificant differences are hatched (see methods section). This Figure is produced using Cartopy which is licensed under the GNU Lesser General Public License.

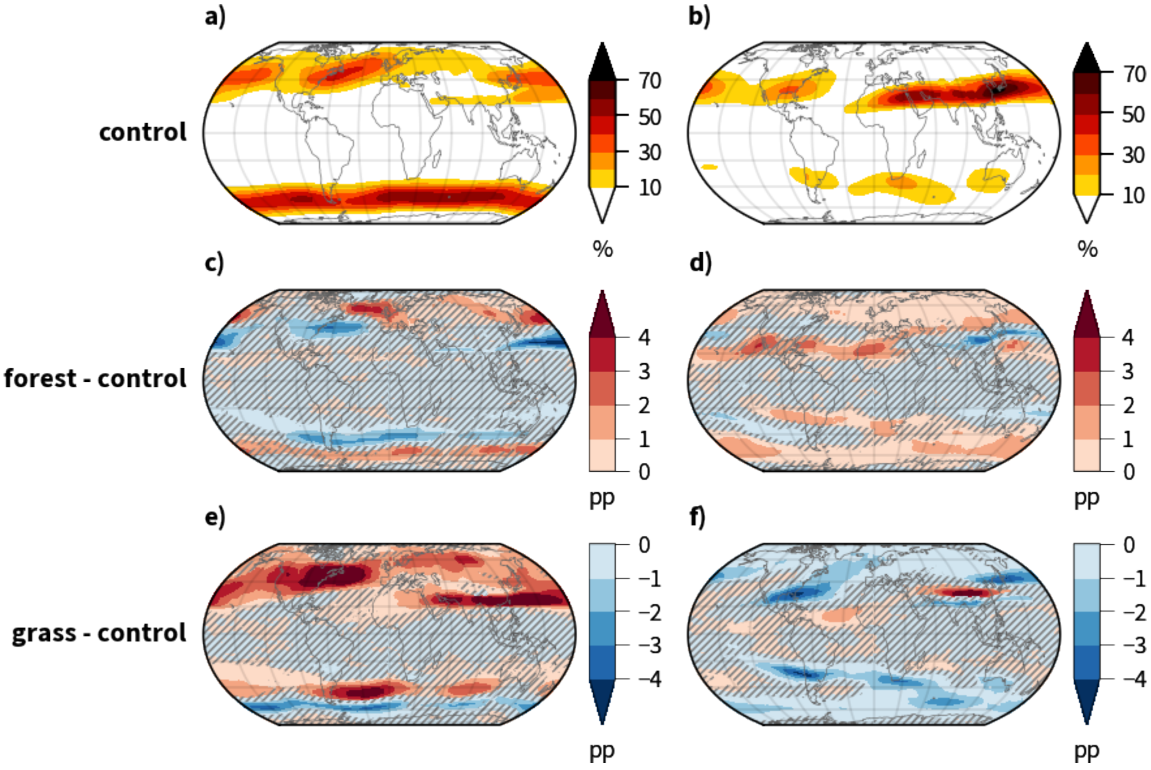

Supplementary Figure 16: Jet frequency changes in boreal winter. Mean October to March frequencies of (a,c,e) deep jets and (b,d,f) shallow jets in (a,b) control (shading, in %) and (c,d) differences in forest and (e,f) differences in grass relative to control. Statistically insignificant differences are hatched (see methods section). This Figure is produced using Cartopy which is licensed under the GNU Lesser General Public License.

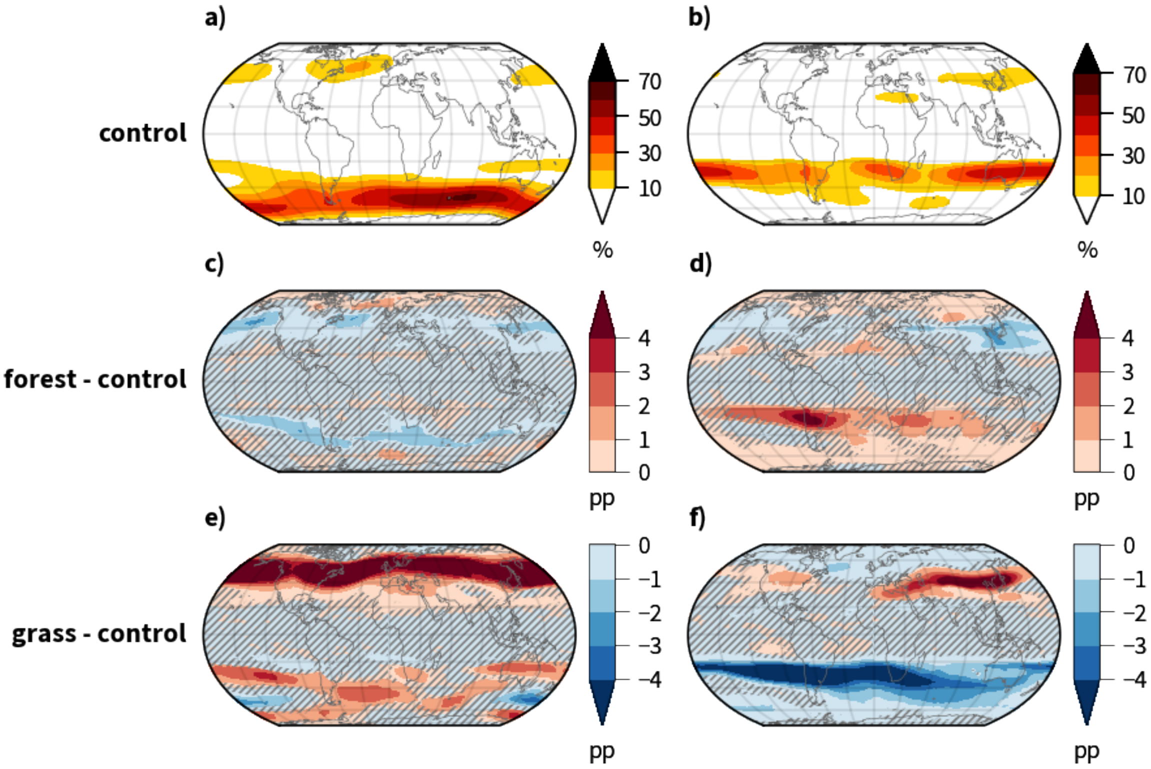

Supplementary Figure 17: Jet frequency changes in boreal summer. Mean April to September frequencies of (a,c,e) deep jets and (b,d,f) shallow jets in (a,b) control (shading, in %) and (c,d) differences in forest and (e,f) differences in grass relative to control. Statistically insignificant differences are hatched (see methods section). This Figure is produced using Cartopy which is licensed under the GNU Lesser General Public License.

1.4 Hadley cell strength, ITCZ shift, and the subtropical jet stream

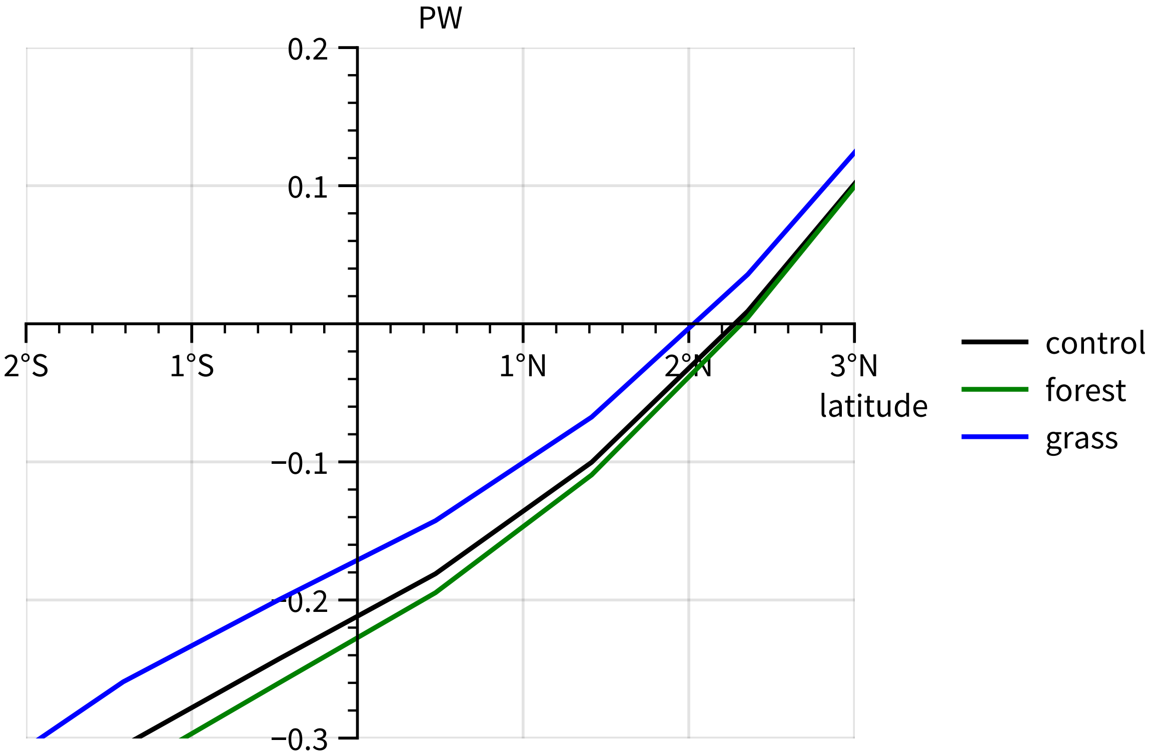

Supplementary Figure 18: Southward shift of the energy flux equator in *grass*. Meridional atmospheric energy flux. The intersection of each line with the 0 PW line marks the position of the energy flux equator.

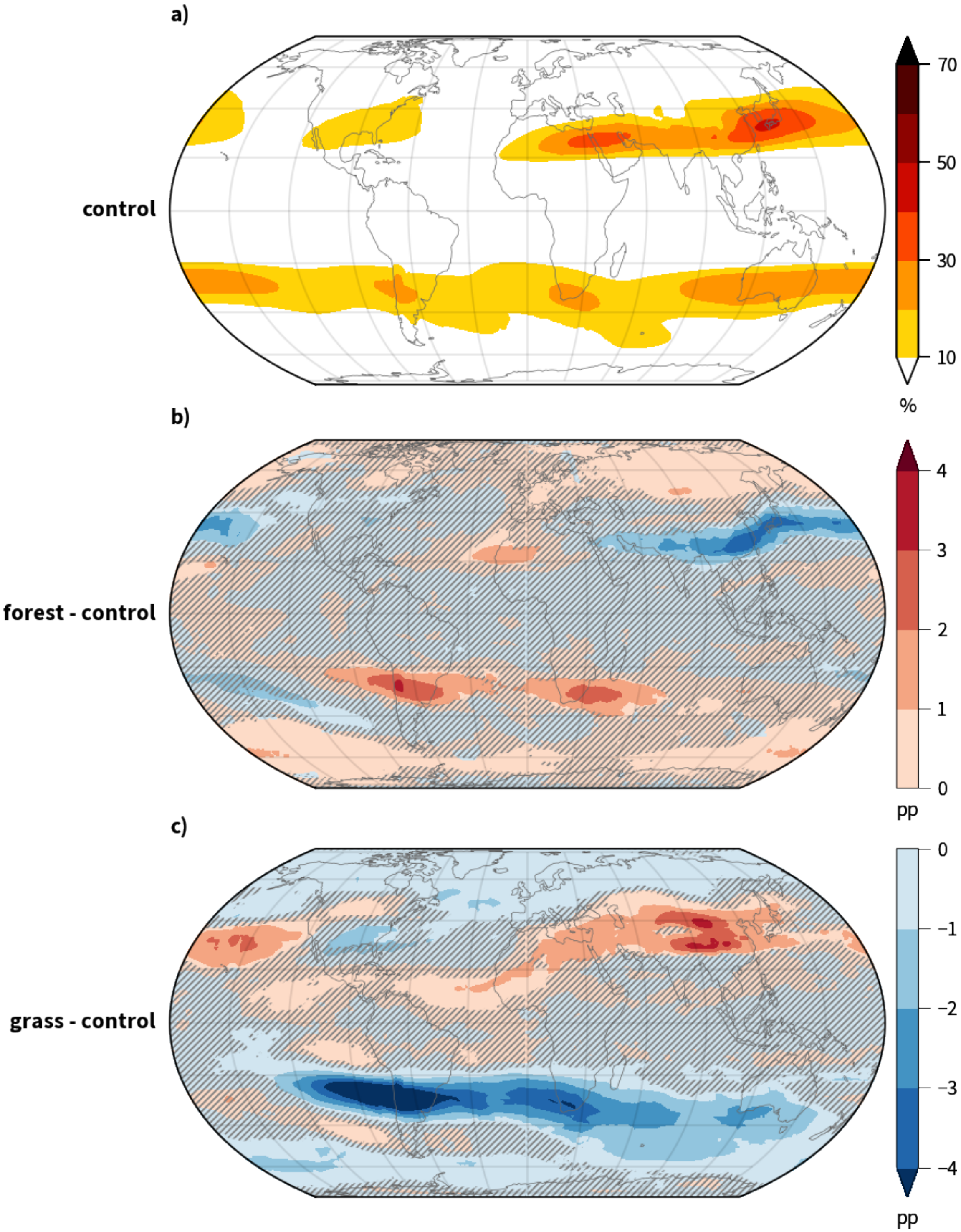

Supplementary Figure 19: Changes in the strength of the subtropical jet streams. Frequencies of shallow jets in (a) *control* (shading, in %) and (b,c) differences with respect to *control* for (b) *forest* and (c) *grass* (in percent points [pp]). Statistically insignificant differences are hatched (see methods section). This Figure is produced using Cartopy which is licensed under the GNU Lesser General Public License.

2 Supplementary Methods

2.1 Atlantic meridional overturning circulation in potential density coordinates

To compute the AMOC in potential density coordinates from the history files of POP, we used an open-access jupyter notebook from github [1]. We applied this computation to each monthly POP history file and then computed annual means.

2.2 Computation of ocean heat content

We compute the ocean heat content within a depth layer from the POP history files according to the following equation

$$H = C_p \int_{z1}^{z2} \rho(z) T(z)\, dz \tag{1}$$

which is as in e.g. [2] but with depth dependent density of sea water $\rho(Z)$. For the specific heat capacity of sea water we used C_p=3996 J (kg K)$^{-1}$. T(z) is the temperature at depth z.

2.3 Computation of the Rossby wave source

We computed the Rossby wave source at 300 hPa according to [3], as also used in [5] and used the open-access code from Windshparm [4] for the computation.

Supplementary References

[1] Stephen G Yeager, POP_MOC (2022), GitHub repository, https://github.com/sgyeager/POP_MOC/blob/main/notebooks/pop_MOCsig2_1deg.ipynb

[2] Roberts, C. D., Palmer, M. D., Allan, R. P., Desbruyeres, D. G., Hyder, P., Liu, C., and Smith, D. (2017), Surface flux and ocean heat transport convergence contributions to seasonal and interannual variations of ocean heat content, J. Geophys. Res. Oceans, 122, 726– 744, doi:10.1002/2016JC012278

[3] Sardeshmukh, P. D., Hoskins, B. J. (1988). The Generation of Global Rotational Flow by Steady Idealized Tropical Divergence, Journal of Atmospheric Sciences, 45, 1228-1251. https://journals.ametsoc.org/view/journals/atsc/45/7/1520-0469_1988_045_1228_tgogrf_2_0_co_2.xml

[4] Dawson, A. (2016). Windspharm: A High-Level Library for Global Wind Field Computations Using Spherical Harmonics. Journal of Open Research Software, 4, e31: http://doi.org/10.5334/jors.129,
Rossby wave source: https://ajdawson.github.io/windspharm/latest/examples/rws_standard.html

[5] Nie, Y., Zhang, Y., Yang, X.-Q., Ren, H.-L. (2019). Winter and summer Rossby wave sources in the CMIP5 models. Earth and Space Science, 6, 1831– 1846. https://doi.org/10.1029/2019EA000674
